# Grooming Coercion and the Post-Conflict Trading of Social Services in Wild Barbary Macaques

**DOI:** 10.1371/journal.pone.0026893

**Published:** 2011-10-26

**Authors:** Richard McFarland, Bonaventura Majolo

**Affiliations:** School of Psychology, University of Lincoln, Brayford Pool, Lincoln, United Kingdom; Yale University, United States of America

## Abstract

In animal and human societies, social services such as protection from predators are often exchanged between group members. The tactics that individuals display to obtain a service depend on its value and on differences between individuals in their capacity to aggressively obtain it. Here we analysed the exchange of valuable social services (i.e. grooming and relationship repair) in the aftermath of a conflict, in wild Barbary macaques (*Macaca sylvanus*). The relationship repair function of post-conflict affiliation (i.e. reconciliation) was apparent in the victim but not in the aggressor. Conversely, we found evidence for grooming coercion by the aggressor; when the victim failed to give grooming soon after a conflict they received renewed aggression from the aggressor. We argue that post-conflict affiliation between former opponents can be better described as a trading of social services rather than coercion alone, as both animals obtain some benefits (i.e. grooming for the aggressor and relationship repair for the victim). Our study is the first to test the importance of social coercion in the aftermath of a conflict. Differences in competitive abilities can affect the exchange of services and the occurrence of social coercion in animal societies. This may also help explain the variance between populations and species in their social behaviour and conflict management strategies.

## Introduction

The exchange or trading of social services between con-specifics (e.g. food, agonistic support or grooming) is a consistent feature of human and animal societies [1]–[5]. Differences in dominance and resource-holding-potential (RHP, e.g. in terms of fighting abilities; [6]) between individuals are expected to play a role on such exchange [3], [4]. Individuals with a higher RHP (usually dominants) can obtain a benefit from subordinates through coercion by using aggression, for example, to prevent other animals from displaying behaviours that may have negative consequences to their own fitness [7]. However, very little is known about how animals exchange social services when differences between individuals exist in their capacity to obtain, give or retain a resource. For example, evidence for social coercion mainly comes from studies focusing on males forcing females to provide social commodities such as grooming or mating opportunities [8], [9], despite coercion potentially affecting any social interaction between two or more individuals differing in their RHP.

The aftermath of a conflict represents an excellent context to explore the trading of social commodities between former opponents and how differences in their RHP modulate such trading [10], [11]. In the first few minutes after a conflict takes place, former opponents are more at risk of receiving aggression from one another or from third parties [12]. This results in a period of social uncertainty which can cause a dramatic increase in the anxiety of the opponents ([12], [13], in our study population: [14]). Reconciliation (the friendly interaction between former opponents in the minutes immediately following a conflict; [12]) functions to reduce the risk of renewed aggression and the opponent's post-conflict anxious response [15]. Reconciliation also repairs the opponent's social relationship disrupted by the conflict; a function that is particularly beneficial for opponents sharing a high quality relationship [16], [17]. This is because these individuals would incur higher costs (in terms of damage to their relationship and its associated benefits for individual fitness; [18]) if reconciliation does not take place, compared to opponents that share low quality relationships (as these individuals have less to lose if these relationships are damaged).

The costs of a conflict and the benefits of reconciling it, however, differ according to the role of the opponents. When directly compared, the risk of receiving renewed aggression and the increase in anxiety have been found to be significantly higher for the victim (usually a subordinate individual) than the aggressor (i.e. a dominant individual) in the aftermath of a conflict [14], [16], [19]. The differential costs experienced by the opponents are also expected to affect their behaviour in the aftermath of a conflict. For example, the higher the post-conflict anxious response of an animal, the less likely they are to reconcile [20]. Similarly, the benefits associated with the relationship repair function of reconciliation may differ between the victim and the aggressor. In many mammalian societies, for example, dominant individuals have a stronger and larger network of friendly relationships with other group members than subordinates do [4], [21]. Therefore, the costs associated with failing to reconcile a conflict, which may in turn determine the opponent's post-conflict behaviour, will depend on the relative value that the victim and aggressor pose on their social relationship with each other. Such relative value can be measured as the ratio of the relationship quality shared by two animals to the baseline quality of friendly relationships that each animal shares with other group members.

Our aim was to analyse, in wild Barbary macaques (*Macaca sylvanus*), the post-conflict trading of two valuable social services between former opponents: the repair of social relationships and post-conflict grooming. Grooming is a valuable service as it provides a number of benefits to the recipient (e.g. endorphin release: [22], anxiety reduction [23], [24]) and costs to the donor (e.g. reduced vigilance; [25]). Moreover, grooming is the most common behaviour primates use to reconcile and maintain friendly social relationships [26].

Based on the costs of aggression (i.e. renewed aggression and increased anxiety) for the victim but not for the aggressor in our study population [14], and on the fact that in primates, and especially in macaques, dominant individuals have a stronger and larger network of grooming interactions with their group companions than subordinates [21], we aimed to test the following predictions. First, we predicted that the benefits of relationship repair with the former opponent after a conflict would be higher for victims of aggression than for aggressors. If this is so, the occurrence of reconciliation should be affected by the relative quality that a social relationship has for the victim, but not for the aggressor. Second, we predicted that aggression and renewed aggression would be used by the aggressor to coerce a valuable service (i.e. grooming) from the victim. This would result in the aggressor being more likely to initiate reconciliation than the victim, receiving more post-conflict grooming than the victim, and at higher proportions than baseline levels. Moreover, we predicted that the aggressor should approach and try to receive grooming from the victim after a conflict and the aggressor should be aggressive towards the victim if they fail to give grooming.

## Methods

### a) Ethics statement

This study complies with Moroccan and UK regulations regarding the ethical treatment of research subjects. Permission to conduct the study was given by the Ethics Committee of the University of Lincoln, UK and by the Haut Commissariat des Eaux et Forêts, Morocco (no permission IDs were given). The study was fully observational and our data collection did not affect the monkeys' welfare.

### b) Study subjects

The Barbary macaque is a terrestrial primate in which grooming is frequently exchanged and group members arrange themselves according to a clear dominance hierarchy [27]. Subjects of this study were 48 adult or sub-adult monkeys (30 males and 18 females) living in two groups of wild Barbary macaques. These two groups (named ‘Flat-face’ and ‘Large’ group) inhabited the deciduous cedar-oak forest near the city of Azrou (33° 24′N–005° 12′W), in the Middle-Atlas Mountains of Morocco. At the beginning of the study, the ‘Flat-face’ group consisted of 29 individuals (10 adult males, 1 sub-adult male, 8 adult females, 5 juveniles and 5 infants) and the ‘Large’ group consisted of 39 individuals (16 adult males, 3 sub-adult males, 10 adult females, 7 juveniles and 3 infants).

### c) Data collection

Data were collected daily between 06.00 and 19.00 hours from June 2008 to September 2009. When a conflict was observed between two or more monkeys, data were collected on the identity of the animals and their role in the conflict (i.e. aggressor or victim, the aggressor being defined as the initiator of the first aggressive display and the victim as the recipient of this aggression). The role of the monkeys in a conflict reflected their dominance relationships, as the aggressor was dominant over the victim in 96% of the conflicts observed. Counter-aggression (i.e. a victim being aggressive towards the former aggressor) was only observed in 4% of conflicts. Data were also collected on the number of opponents involved in the conflict.

After a conflict was over (i.e. no aggression was observed for at least 30 seconds), focal data were recorded from the victim or aggressor for five minutes as this time window encapsulates the peak of post-conflict social interaction [12], [14]. During post-conflict focal sessions we recorded the timing and occurrence of any aggressive (i.e. threat, lunge, charge, chase, slap, grab or bite) or friendly interaction between our focal animal and any other group member. We considered successful ≤1.5 metre approaches (i.e. approaches that were not followed by aggression or displacement for the first 30 seconds after the approach), allo-grooming, body-contact and teeth-chattering as affiliative behaviour [28]. When grooming was observed between former opponents in the post-conflict period we recorded the duration and direction of grooming exchanged until the grooming session was terminated (i.e. no grooming was observed for at least 30 seconds), even if this lasted beyond the set five minute focal session. We used the same rule when collecting focal samples (see below).

We used focal and scan sampling to collect data on the baseline affiliation of each dyad and on their relationship quality. Scan samples were collected every hour on the activity of the study animals (i.e. allo-grooming, body contact, feeding, resting), their ≤1.5 metre proximity to other study subjects, and on the identity of their social partners. Moreover, we collected 20 minute continuous baseline focal sessions on our study animals to calculate the proportion of successful ≤1.5 metre approaches, the direction and duration of grooming exchanged within each dyad. For each animal, focal observations were evenly distributed across the study period and time of day.

### d) Data analysis

Analyses were based on data from 414 post-conflict focal observations collected from the victim (N = 191) and the aggressor (N = 223). All but one adult male of the ‘Large’ group, and all of the study monkeys from the ‘Flat-face’ group were represented in at least one post-conflict session (mean post-conflict sessions per monkey ± SE = 17.6±2.2). Moreover, we also collected 792 scan samples and 1,101.9 hours of baseline focal observations (mean hours/monkey ± SE = 18.71±2.10).

A conflict was considered to be reconciled when friendly behaviour (i.e. body-contact, teeth-chattering or grooming) was exchanged between the former opponents within five minutes of the conflict [12]. We also considered close-proximity approaches as a form of reconciliation as there is evidence that close proximity functions to reconcile in the Barbary macaque [14], [29]. For example, post-conflict close-proximity approaches reduce anxiety even if they are not followed by grooming (McFarland & Majolo, in preparation). In order to test if differences in the importance two animals pose on their relationship with each other affect their post-conflict behaviour, we calculated a measure of relationship quality for each member of a dyad. For a given dyad composed of monkey A and B, we calculated the relationship quality between A and B following the formula [18]:



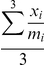




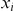
 =  Individual's mean value for each of the three behavioural measures.


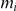
 =  Group's mean value for each of the three behavioural measures.

To calculate 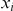 we first obtained a mean value per monkey by collapsing together the average proportion of hourly scans in which A and B were exchanging friendly behaviour or were within ≤1.5 metre proximity, and the proportion of successful ≤1.5 metre approaches exchanged between them, collected during the 20 minute focal sessions. This figure was divided by the total proportion of friendly behaviour, proximity and approaches each monkey exchanged with all other group members. The same three variables were used to calculate medians at the group level to obtain 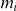. Following these calculations, the value of the composite sociality index obtained for each monkey measured the relative importance that a social relationship between monkey A and B had for monkey A (or B) with respect to the overall quality of the relationships monkey A (or B) shared with other group members. Moreover, the composite sociality index controlled for the overall affiliation at the group level which was important when analysing data coming from two different groups. The higher the value of the composite sociality index, the stronger the relationship quality was for monkey A (or B).

Data on the average duration of grooming exchanged between dyads were extracted from the baseline focal sessions. The percentage of grooming received by monkey A (or B) in a grooming bout (from post-conflict or baseline observations) was calculated using the following equation: (grooming received by A / grooming received by A + grooming received by B) x 100. Data were analysed using a series of generalised linear mixed models (GLMMs). GLMMs allow analysing the effect of a series of independent variables (i.e. fixed factors) on a continuous or categorical variable [30]. Moreover, GLMMs allowed us to run the analyses using each post-conflict session as a single data point through the inclusion of random factors to the models [30]. In the GLMMs we thus entered victim and aggressor ID as random factors to control for the non-independence of multiple data coming from the same subjects. The occurrence of reconciliation (i.e. yes or no), percentage of post-conflict grooming received, and frequency of post-conflict aggression were our dependent variables in a series of GLMMs.

To test our predictions, we entered as fixed factors, respectively, the role of opponents (i.e. aggressor or victim), the relative measure of relationship quality for the aggressor and the victim, condition (post-conflict or baseline observation), and whether a post-conflict approach was followed by grooming or not. Moreover, as ‘control’ fixed factors we entered group ID (i.e. ‘Flat-face’ or ‘Large’ group), age combination of the dyad (i.e. adult-adult, subadult-subadult or adult-subadult), their sex combination (i.e. male-male, female-female or male-female), their rank distance, and ‘bystander affiliation’ (i.e. whether or not the focal animal exchanged a friendly interaction with a bystander in the post-conflict session) because these variables can affect post-conflict behaviour and/or mediate the costs of aggression [15], [31].

Below we present the results for our predicting fixed factors. Results for the ‘control’ fixed factors are shown in the electronic supporting information attached to this manuscript (see Tables S1, S2, S3, S4, S5, S6). GLMM data analyses were performed using STATA v10.1 Software [32]. When comparing the proportion of reconciliatory events initiated by the victim or aggressor, a chi-square test was performed using PASW Statistics v17.

## Results

**Figure 1:**
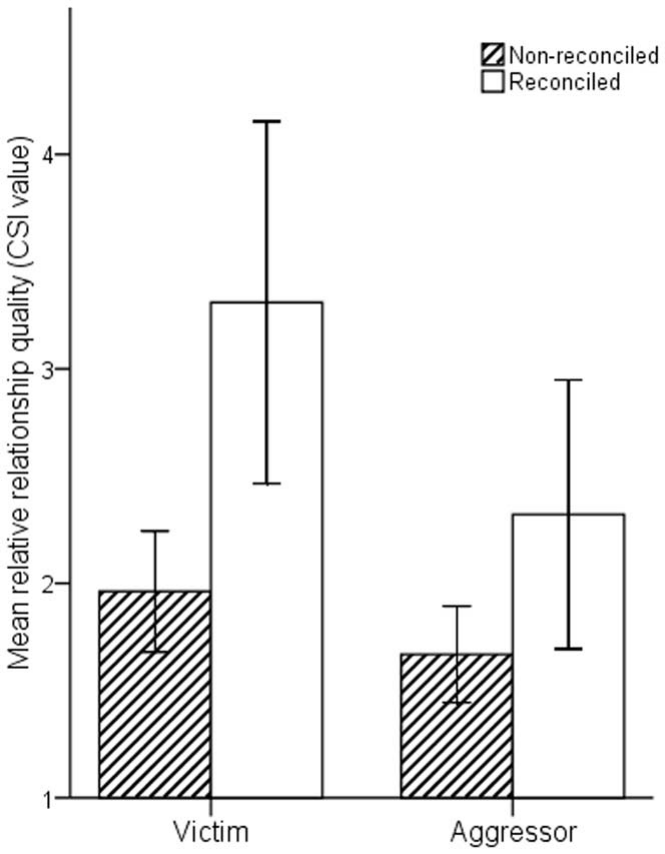
Histogram showing the opponent's relationship quality (composite sociality index) from the perspective of the victim and aggressor for reconciled and non-reconciled conflicts.

In support of our first prediction, the occurrence of reconciliation was significantly predicted by the relative quality that a social relationship had for the victim (GLMM, β ± SE = 0.02±0.01, z = 2.82, N = 414, p<0.01; Figure 1 and Table S1), but not for the aggressor (β ± SE = 0.01±0.01, z = 0.65, N = 414, p = 0.52; Figure 1).

**Figure 2:**
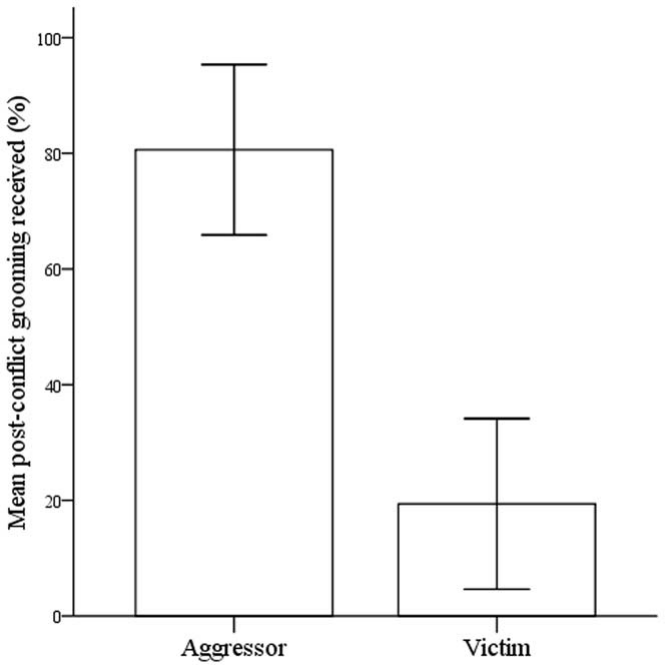
Histogram showing the proportion of post-conflict grooming received by the victim and aggressor.

**Figure 3:**
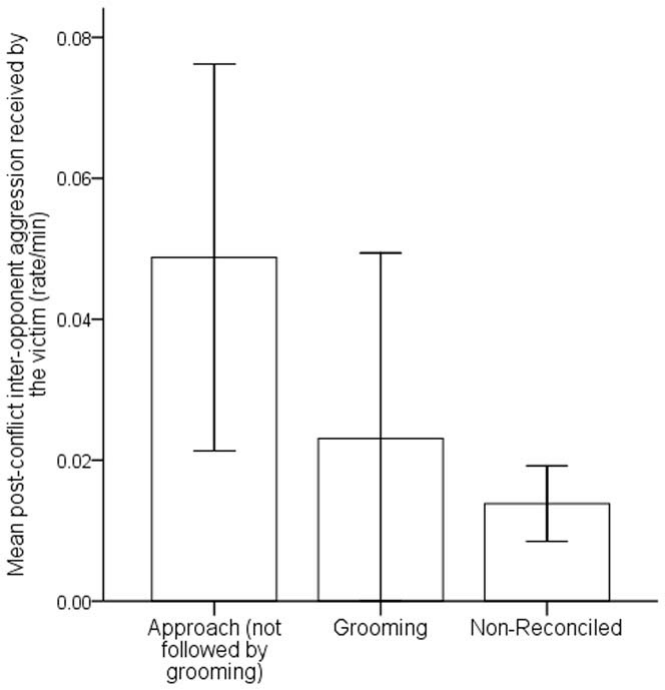
Histogram showing the mean rate of inter-opponent aggression received by the victim following post-conflict approaches or grooming and non-reconciled conflicts.

In support of our second prediction, the aggressor initiated reconciliation significantly more often than the victim (aggressor  = 63%, victim  = 37%; chi-square test, χ2 (1, N = 65)  = 4.45, p<0.05). The aggressor received a significantly larger percentage of post-conflict grooming than the victim (β ± SE = −61.23±10.55, z = −5.80, N = 52, p<0.001; Figure 2 and Table S2). Moreover, the aggressor received a significantly larger percentage of grooming in the post-conflict period (mean ± SE = 80.61%±7.15) compared to baseline levels (mean ± SE = 71.17%±5.74; β ± SE = 9.44±4.39, z = −2.33, N = 52, p<0.05; Table S3). Conversely, the victim received a significantly smaller proportion of grooming in the post-conflict period (mean ± SE = 19.39%±7.16) compared to baseline levels (mean ± SE = 28.83%±5.75; β ± SE = −9.44±4.05, z = −2.33, N = 52, p<0.05; Table S4). Finally, the victim received significantly more aggression from the aggressor when a post-conflict ≤1.5 metre approach was not followed by grooming compared to non-reconciled conflicts (β ± SE = 0.04±0.01, z = 3.86, N = 382, p<0.001; Figure 3 and Table S5). We found no such difference in aggression when a post-conflict ≤1.5 metre approach was followed by grooming in comparison to non-reconciled conflicts (β ± SE = 0.01±0.01, z = 0.63, N = 408, p = 0.53; Figure 3 and Table S6).

## Discussion

Our results indicate that the post-conflict behaviour of the victim and aggressor is affected by the services at stake. From the victim's perspective, a social relationship is more likely to be repaired the higher the relative value of their relationship quality with the former aggressor. This supports the hypothesis that more valuable relationships are more frequently reconciled [12] due to the fitness-related benefits of maintaining friendly relationships with other group members [18]. The greater benefit to the victim in repairing a damaged social relationship, compared to the aggressor, became apparent when considering the relative value the opponents pose on their social relationship and the higher costs of aggression to the victim [14], [16], [19]. Moreover, in our study population post-conflict close-proximity approaches and grooming reduce the victim's anxiety [14]. Alternatively, from the aggressor's perspective, the aftermath of a conflict represents an apparent ‘opportunity’ to obtain grooming from the former victim by taking advantage of the emotional cost of aggression and the stronger need to reconcile for the victim [12], [14]. The aggressor initiated reconciliation more often and received significantly more post-conflict grooming than the victim. Moreover, the aggressor received proportionally more post-conflict grooming from the victim than baseline levels. Finally, when a post-conflict close-proximity approach (which functions to reconcile a conflict in the current study population; 14) was not followed by grooming, the victim had a higher risk of receiving renewed aggression.

Overall, our findings show that the post-conflict behaviour of Barbary macaques may be described by two, non-mutually exclusive phenomena: the trading of social services (i.e. grooming for the aggressor and relationship repair plus reduced anxiety [14] for the victim) and the coercion of grooming. Social primates, including cercopithecines, have been shown to effectively use information about the quality of con-specific social relationships to their own advantage, for example when choosing coalition partners [33]. Moreover, primates seem to be capable of displaying complex social tactics such as tactical deception [34]. As such, the capacity for macaques to coerce social services in the aftermath of a conflict and to display flexible social tactics based on the benefits and costs at stake appears theoretically possible. The trading of social services and grooming coercion, however, does not necessarily require complex cognitive abilities; the emotional state of the victim may drive their post-conflict behaviour [20], [35]. Moreover, reconciliation is considered a low cognitively demanding mechanism [12]. It is more difficult to explain the behaviour of the aggressor according to their emotional state as no post-conflict increase of anxiety was found in the aggressor [14]. However, it is possible that the behaviour of the aggressor was elicited by observing the behavioural manifestation of anxiety in the victim without necessarily implying any empathic response in the aggressor [12].

Conclusive evidence for coercion in animal societies is scarce [7] whereas this is a common feature of resource exchange in humans [3]. The results of our study suggest that the coercion of social commodities may be an important factor affecting social exchanges in animal societies. We argue that the possibility of coercion as a result of differences in RHP should be more explicitly incorporated into current theoretical frameworks analysing service exchange in animals, such as the biological market approach [1] and the different types of reciprocity [36]. For example, the fact that grooming is mainly directed to dominant individuals in primate societies [21] is usually explained by the more valuable services they can offer (due to their higher RHP), which makes dominants the best grooming partners. However, grooming coercion from dominants to subordinates may contribute to the network of grooming exchange in a group [9]. If so, differences between species in the degree of grooming bias towards dominant individuals [37] may be modulated by species-specific differences in the ‘capacity’ (in terms of RHP differences between social partners) or ‘opportunity’ to coerce and not only by differences in the social value of grooming.

Two important conclusions can be drawn from our findings. First, studies of social relationships should take into account the relative importance of a relationship to the two members of a dyad. This is essential to better our understanding of the mechanisms behind social exchange and to make predictions on what social tactics are beneficial for individual fitness. Indeed, social relationships are expected to be ‘intrinsically’ asymmetric due to differences in RHP and dominance between social partners, which affects the type and frequency of behaviours exchanged. For example, primates exchange grooming for other commodities (e.g. tolerance near food) and the asymmetric distribution of this exchange is reflected in the relative dominance of social partners (i.e. dominant individuals can trade tolerance for grooming; [2]). However, studies of social behaviour and conflict management have often analysed relationship quality at the dyadic level [16], [17].

Secondly, we conclude that the trading or exchange of social commodities in the aftermath of a conflict should be considered in studies of conflict management. We are not suggesting here that every aggressive interaction should be viewed as an act of coercion. However, we do suggest that the occurrence of such exchanges and the differential costs of aggression to opponents, due to their differences in RHP, need to be carefully analysed in studies of conflict management. This is especially important when making comparisons across populations, species or higher taxonomic levels. For example, the trading of social commodities could help explain the variability in conflict management strategies found within and between dyads, as well as across populations or species [12].

## Supporting Information

Table S1 Results of GLMM for the relationship between the occurrence of reconciliation and opponent relative relationship quality
(DOC)

Table S2 Results of GLMM for the relationship between post-conflict grooming received and opponent identity (i.e. aggressor or victim)
(DOC)

Table S3 Results of GLMM for the relationship between the percentage of grooming received by the aggressor and PC-baseline session
(DOC)

Table S4 Results of GLMM for the relationship between the percentage of grooming received by the victim and PC-baseline session
(DOC)

Table S5 Results of GLMM for the relationship between the amount of inter-opponent aggression received by the victim and PC close-proximity approaches (excluding grooming)
(DOC)

Table S6 Results of GLMM for the relationship between the amount of inter-opponent aggression received by the victim and PC grooming
(DOC)
