# Supplementary material for: Grooming Coercion and the Post-Conflict Trading of Social Services in Wild Barbary Macaques
Source: PLoS One. 2011 Oct 26;6(10):e26893. doi: 10.1371/journal.pone.0026893 (PMC3202593; doi:10.1371/journal.pone.0026893)
Supplement: Table S1 — Results of GLMM for the relationship between the occurrence of reconciliation and opponent relative relationship quality (DOC) [file pone.0026893.s001.doc]

Table S1. Results of GLMM for the relationship between the occurrence of reconciliation and opponent relative relationship quality

|  | β ± SE | Z | P | N | 95% CIs |
| --- | --- | --- | --- | --- | --- |
| Group | -0.11 ± 0.05 | -2.38 | 0.02 | 414 | -0.20 – -0.02 |
| Age combination | -0.03 ± 0.03 | -1.10 | 0.27 | 414 | -0.10 – 0.03 |
| Sex combination | 0.01 ± 0.04 | 0.23 | 0.82 | 414 | -0.07 – 0.09 |
| Rank difference | 0.01 ± 0.00 | 2.54 | 0.01 | 414 | 0.00 – 0.02 |
| Victim’s relative  relationship quality | 0.02 ± 0.01 | 2.82 | 0.01 | 414 | 0.01 – 0.04 |
| Aggressor’s relative  relationship quality | 0.01 ± 0.01 | 0.65 | 0.52 | 414 | -0.01 – 0.03 |
