# Supplementary material for: Grooming Coercion and the Post-Conflict Trading of Social Services in Wild Barbary Macaques
Source: PLoS One. 2011 Oct 26;6(10):e26893. doi: 10.1371/journal.pone.0026893 (PMC3202593; doi:10.1371/journal.pone.0026893)
Supplement: Table S2 — Results of GLMM for the relationship between post-conflict grooming received and opponent identity (i.e. aggressor or victim) (DOC) [file pone.0026893.s002.doc]

Table S2. Results of GLMM for the relationship between post-conflict grooming received and opponent identity (i.e. aggressor or victim)

|  | β ± SE | Z | P | N | 95% CIs |
| --- | --- | --- | --- | --- | --- |
| Group | -0.00 ± 18.03 | -0.00 | 1.00 | 52 | -35.34 – 35.34 |
| Age combination | -0.00 ± 22.01 | 0.00 | 1.00 | 52 | -43.14 – 43.14 |
| Sex combination | -0.00 ± 17.26 | 0.00 | 1.00 | 52 | -34.63 – 36.63 |
| Rank difference | -0.00 ± 10.55 | -0.00 | 1.00 | 52 | -2.86 – 2.86 |
| Opponent ID | -61.22 ± 10.55 | -5.80 | <0.001 | 52 | -81.90 – -40.55 |
