# Supplementary material for: Grooming Coercion and the Post-Conflict Trading of Social Services in Wild Barbary Macaques
Source: PLoS One. 2011 Oct 26;6(10):e26893. doi: 10.1371/journal.pone.0026893 (PMC3202593; doi:10.1371/journal.pone.0026893)
Supplement: Table S3 — Results of GLMM for the relationship between the percentage of grooming received by the aggressor and PC-baseline session (DOC) [file pone.0026893.s003.doc]

Table S3. Results of GLMM for the relationship between the percentage of grooming received by the aggressor and PC-baseline session

|  | β ± SE | Z | P | N | 95% CIs |
| --- | --- | --- | --- | --- | --- |
| Group | 35.13 ± 17.92 | 1.96 | 0.05 | 52 | 0.02 – 70.25 |
| Age combination | -15.00 ± 22.08 | -0.58 | 0.50 | 52 | -58.57 – 28.27 |
| Sex combination | -18.89 ± 14.51 | -1.30 | 0.19 | 52 | -47.34 – 9.55 |
| Rank difference | -2.54 ± 1.27 | -2.00 | 0.04 | 52 | -5.03 – -0.05 |
| PC-Baseline | -9.44 ± 4.05 | -2.33 | 0.02 | 52 | -17.39 – -1.49 |
