# Supplementary material for: Grooming Coercion and the Post-Conflict Trading of Social Services in Wild Barbary Macaques
Source: PLoS One. 2011 Oct 26;6(10):e26893. doi: 10.1371/journal.pone.0026893 (PMC3202593; doi:10.1371/journal.pone.0026893)
Supplement: Table S4 — Results of GLMM for the relationship between the percentage of grooming received by the victim and PC-baseline session (DOC) [file pone.0026893.s004.doc]

Table S4. Results of GLMM for the relationship between the percentage of grooming received by the victim and PC-baseline session

|  | β ± SE | Z | P | N | 95% CIs |
| --- | --- | --- | --- | --- | --- |
| Group | 35.13 ± 17.92 | -1.96 | 0.05 | 52 | -70.25 – -0.02 |
| Age combination | -15.00 ± 22.08 | 0.58 | 0.50 | 52 | -28.27 – 58.27 |
| Sex combination | -18.89 ± 14.51 | 1.30 | 0.19 | 52 | -9.55 – 47.34 |
| Rank difference | -2.54 ± 1.27 | 2.00 | 0.04 | 52 | 0.05 – 5.03 |
| PC-Baseline | -9.44 ± 4.05 | 2.33 | 0.02 | 52 | 1.49 – 17.39 |
