# Supplementary material for: Grooming Coercion and the Post-Conflict Trading of Social Services in Wild Barbary Macaques
Source: PLoS One. 2011 Oct 26;6(10):e26893. doi: 10.1371/journal.pone.0026893 (PMC3202593; doi:10.1371/journal.pone.0026893)
Supplement: Table S5 — Results of GLMM for the relationship between the amount of inter-opponent aggression received by the victim and PC close-proximity approaches (excluding grooming) (DOC) [file pone.0026893.s005.doc]

Table S5. Results of GLMM for the relationship between the amount of inter-opponent aggression received by the victim and PC close-proximity approaches (excluding grooming)

|  | β ± SE | Z | P | N | 95% CIs |
| --- | --- | --- | --- | --- | --- |
| Group | 0.01 ± 0.01 | 0.60 | 0.55 | 382 | -0.01 – 0.02 |
| Age combination | 0.00 ± 0.01 | 0.45 | 0.65 | 382 | -0.01 – 0.01 |
| Sex combination | -0.02 ± 0.01 | -2.55 | 0.01 | 382 | -0.03 – -0.00 |
| Rank difference | 0.00 ± 0.00 | 0.05 | 0.95 | 382 | -0.00 – 0.00 |
| Number of opponents | 0.01 ± 0.01 | 1.22 | 0.22 | 382 | -0.01 – 0.03 |
| Approach (no groom) | 0.04 ± 0.01 | 3.85 | <0.001 | 382 | 0.02 – 0.06 |
| Bystander affiliation | 0.01 ± 0.01 | 2.07 | 0.04 | 382 | 0.00 – 0.03 |
