# Supplementary material for: Grooming Coercion and the Post-Conflict Trading of Social Services in Wild Barbary Macaques
Source: PLoS One. 2011 Oct 26;6(10):e26893. doi: 10.1371/journal.pone.0026893 (PMC3202593; doi:10.1371/journal.pone.0026893)
Supplement: Table S6 — Results of GLMM for the relationship between the amount of inter-opponent aggression received by the victim and PC grooming (DOC) [file pone.0026893.s006.doc]

Table S6. Results of GLMM for the relationship between the amount of inter-opponent aggression received by the victim and PC grooming

|  | β ± SE | Z | P | N | 95% CIs |
| --- | --- | --- | --- | --- | --- |
| Group | 0.00 ± 0.01 | 0.15 | 0.88 | 408 | -0.01 – 0.02 |
| Age combination | 0.00 ± 0.01 | 0.01 | 1.00 | 408 | -0.01 – 0.01 |
| Sex combination | -0.02 ± 0.01 | -2.51 | 0.01 | 408 | -0.03 – -0.00 |
| Rank difference | 0.00 ± 0.00 | 0.77 | 0.44 | 408 | -0.01 – 0.00 |
| Number of opponents | 0.01 ± 0.01 | 0.99 | 0.32 | 408 | -0.01 – 0.03 |
| Grooming | 0.01 ± 0.01 | 0.63 | 0.53 | 408 | -0.02 – 0.03 |
| Bystander affiliation | 0.01 ± 0.01 | 1.65 | 0.10 | 408 | -0.00 – 0.02 |
